# Osteosarcoma of the bladder: a case report

**DOI:** 10.1186/s13256-022-03346-2

**Published:** 2022-03-25

**Authors:** Toshiki Oka, Kyosuke Matsuzaki, Haruka Izumi, Daichi Nonomura, Kiyoshi Mori, Kensaku Nishimura

**Affiliations:** 1 Department of Urology, Osaka National Hospital, Hoenzaka 2-1-14, Osaka, 540-0006 Japan; 2 Department of Pathology, Osaka National Hospital, Osaka, Japan

**Keywords:** Bladder, Osteosarcoma, TURBT, Extraskeletal osteosarcoma, Case report

## Abstract

**Background:**

Osteosarcoma arising from the bladder is extremely rare, with only 38 cases reported to our knowledge. It is often detected owing to hematuria, and is treated by surgery (for example, total cystectomy), radiation therapy, and chemotherapy; however, the prognosis is extremely poor.

**Case presentation:**

An 83-year-old Japanese man underwent cystoscopy for postoperative follow-up of urothelial carcinoma of the bladder, which revealed a 2-cm nodular tumor on the right wall. He had a history of abdominal aortic aneurysm and hypertension, and had been smoking 15 cigarettes per day for 45 years. Seven years previously, the patient underwent transurethral resection of bladder tumor for a 5-cm tumor on the right wall of the bladder. The histopathological diagnosis was urothelial carcinoma. No recurrence had been detected since then. Transurethral resection of bladder tumor was performed, and the histopathological diagnosis was cystosarcoma. Because of his advanced age, we decided that it would be difficult to perform total cystectomy. We therefore performed a second transurethral resection of bladder tumor and found no residual tumor. At 29 months after surgery, the patient remains alive without recurrence.

**Conclusion:**

Bladder osteosarcoma has a poor prognosis. However, our case was detected early, and treatment with transurethral resection of bladder tumor alone resulted in long-term survival without recurrence.

**Supplementary Information:**

The online version contains supplementary material available at https://doi.org/10.1186/s13256-022-03346-2.

## Background

Osteosarcoma arising from the bladder is extremely rare. It is treated by total cystectomy, radiation therapy, and chemotherapy; however, the prognosis is extremely poor. In this article, we report a case of bladder osteosarcoma that was discovered asymptomatically during follow-up of urothelial carcinoma of the bladder. The patient was treated by transurethral resection of bladder tumor (TURBT) alone and achieved long-term survival without recurrence.

## Case presentation

**Fig. 1.**
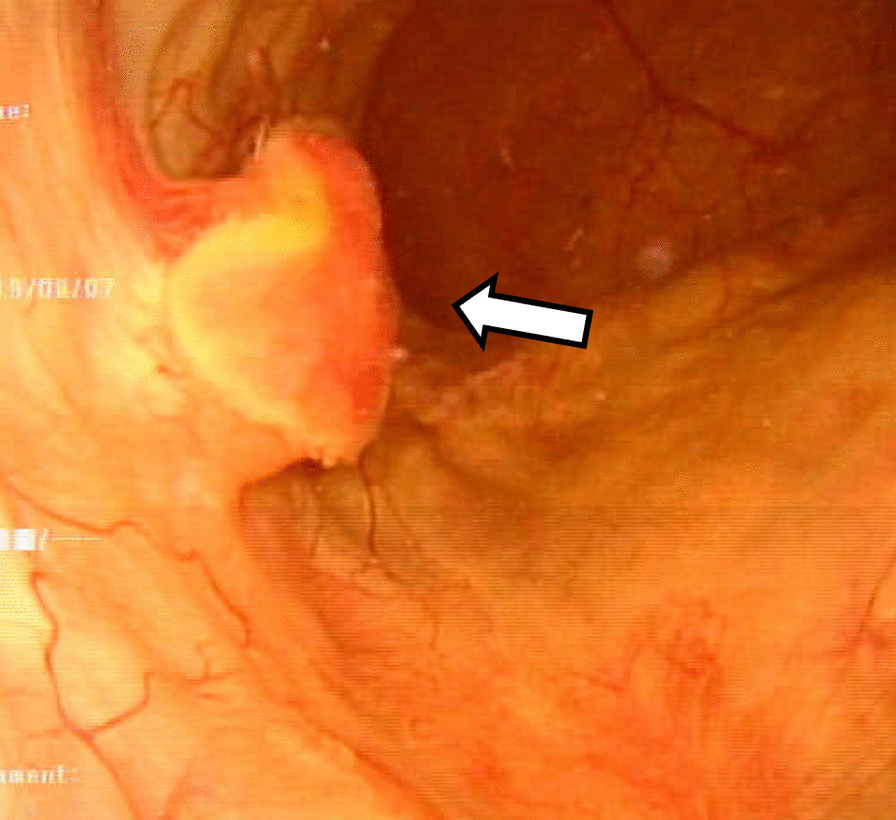
Cystoscopy showing a 2 cm nodular tumor on the right wall. The arrow points to the tumor

**Fig. 2.**
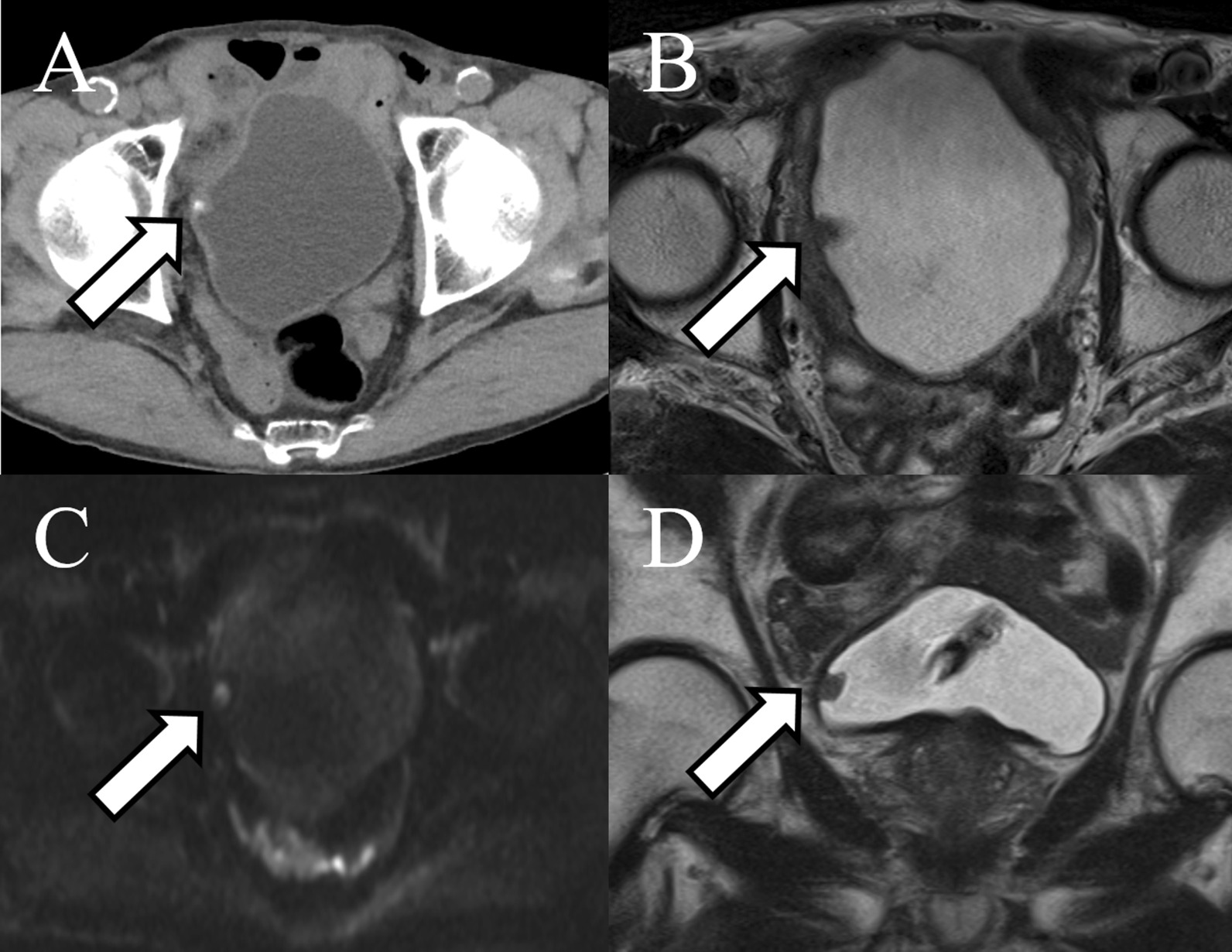
**A** Abdominal computed tomography showing a mass with calcification on the right wall. Magnetic resonance imaging also showed the tumor. **B** Axial section of a T2-weighted image. **C** The tumor showed a high signal intensity on diffusion-weighted imaging. **D** Coronal section of a T2-weighted image. The arrow points to the tumor

**Fig. 3.**
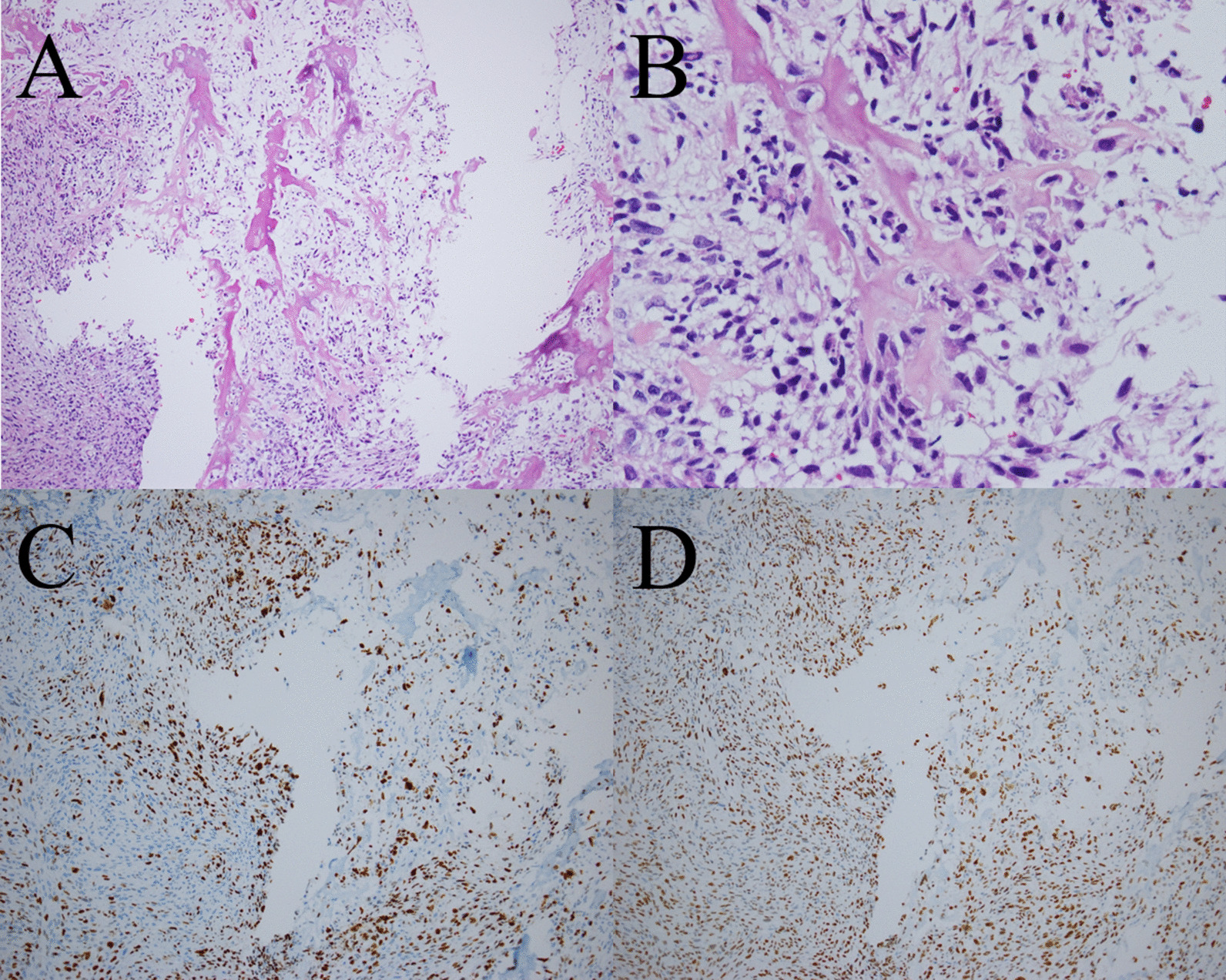
**A**, **B** Microscopic examination of the resected tissue showing spindle-shaped or star-shaped cells with irregularly enlarged nuclei, some of which show unnatural formation of osteoid and cartilage tissue. **A** Low-power view of hematoxylin- and eosin-stained section. **B** High-power view of hematoxylin- and eosin-stained section. **C** Expression of Ki-67 analyzed by immunostaining. The labeling index was 30%. **D** Expression of p53 analyzed by immunostaining, showing diffuse positive images beyond the range of Ki-67 positivity

The patient was an 83-year-old Japanese man. During routine cystoscopy after TURBT, a 2-cm nodular tumor was found on the right wall (Fig. 1). He had a history of abdominal aortic aneurysm and hypertension, and had smoked 15 cigarettes per day for 45 years. In October 2012, he underwent TURBT for a 5-cm bladder cancer on the right wall. The histopathological diagnosis was urothelial carcinoma, G2, pTa. Since then, the patient had undergone regular follow-up examinations and no recurrence had been observed. This time, however, intravesical recurrence was observed; thus, the patient was examined closely. Abdominal computed tomography (CT) showed a mass with calcification in the right wall. On magnetic resonance imaging (MRI), the tumor showed a high signal intensity on diffusion-weighted imaging, with no apparent muscle layer invasion, and the vesical imaging reporting and data system (VI-RADS) score was 1 point (Fig. 2). TURBT was performed in August 2019. Microscopic examination of the resected tissue showed spindle-shaped or star-shaped cells with irregularly enlarged nuclei, some of which showed unnatural formations of osteoid and cartilage tissue (Fig. 3 A/B). Immunostaining was positive for Desmin, SMA, Vimentin, Ki-67, and p53, and negative for CD34, c-kit, AE1/AE3, and p16 (Fig. 3 C/D). Since no urothelial carcinoma component was noted and no tumor was found in the systemic bone, we diagnosed the patient with bladder osteosarcoma. We discussed the treatment plan, including total cystectomy; however, owing to his advanced age and the lack of apparent muscle layer invasion on preoperative MRI, a second TURBT procedure was performed in October 2019. Histopathological examination revealed no malignant findings. At 29 months after surgery, the patient remains alive without recurrence.

## Discussion

Osteosarcomas that arise outside of bone are called extraskeletal osteosarcomas. They account for 4% of all osteosarcomas [1]. Radiation therapy, schistosomiasis, trauma, diverticula, use of chemotherapeutic agents (for example, cyclophosphamide), and frequent urinary tract infections are considered risk factors for primary extraskeletal osteosarcoma, including bladder osteosarcoma [2]. Allan *et al.* described the diagnostic criteria for extraskeletal osteosarcoma, as follows: (1) the presence of a uniform morphological pattern of sarcomatous tissue that excludes the possibility of malignant mesenchymoma, (2) the production of malignant osteoid or bone by the sarcomatous tissue, and (3) the exclusion of an osseous origin [3]. Osteosarcoma arising from the bladder is even rarer, with only 38 cases reported to date [2, 4–9]. The diagnosis of osteosarcoma of the bladder requires the exclusion of sarcomatoid changes of urothelial carcinoma, urothelial carcinoma with ossification, carcinoma with pseudosarcomatoid stromal reaction, malignant fibrous histiocytoma, synovial and epithelial sarcoma, and carcinosarcoma of the bladder [10] . Sarcomatoid carcinoma is diagnosed when a malignant epithelial component is identified, even in the presence of osteoid [7]. An immunohistochemical analysis of sarcomatoid carcinoma is at least focally positive for epithelial markers, cytokeratins, and epithelial membrane antigens. The presence of epithelial tumor cells of carcinoma in nests or clusters adjacent to the sarcoma cells is another feature that supports an epithelial origin [11]. On the other hand, osteosarcoma is immunohistochemically negative for pancytokeratins 7 and 20, epithelial membrane antigens, CD34, and CD68. Vimentin and p53 are strongly expressed [7] . Won *et al.* summarized 31 cases of osteosarcoma of the bladder in 2011 [4] . Since then, to our knowledge, 7 cases have been reported, including our case [2, 5–9], and a total of 38 cases are summarized in Additional file 1. For the reports that are available in English, a history of urothelial carcinoma of the bladder and a detailed prognosis have been added. The median age of the patients is 65.5 (24–86) years; however, the patients are often younger than patients with urothelial carcinoma. Many patients experience hematuria. Ours is the only case of urothelial carcinoma of the bladder that was detected without symptoms. Seven cases had a history of urothelial carcinoma of the bladder (two cases appeared at the same time), and had received treatments such as TURBT and tumor excision. Seven patients had obvious metastasis at the time of the diagnosis, and 24 patients did not have metastasis at the time of the diagnosis. Surgery (for example, total cystectomy or tumor excision) was performed in most cases, and radiation was added in four cases. Chemotherapy was also administered in five cases, but the prognosis was very poor, and many patients developed recurrence and died within 1 year. However, radiation therapy and chemotherapy may also have a certain effect, as there are reports of cases in which the primary tumor and bone metastasis showed a complete response to radiation [12], and cases in which ifosfamide, platinum, and methotrexate resulted in tumor shrinkage [13].There were seven patients who survived for more than 1 year. However, this is the only case in which long-term survival was achieved with TURBT alone. We think that this is due to the early detection of bladder cancer during regular follow-up examinations while it remained asymptomatic. Chitiyo identified four possible causes of the development of bladder osteosarcoma: transformation of the bladder epithelium, transformation of the stroma, transfer of osteoblasts by the bloodstream, and generation of osteoblasts from immature mesenchymal tissue derived from the Wolffian body, the developmental home of the bladder triangle [14]. Five patients had a history of treatment for urothelial carcinoma of the bladder, and treatment for urothelial carcinoma of the bladder may have stimulated the development of osteosarcoma. In addition, another three patients had a history of transurethral resection of the prostate (TURP) [8, 15, 16]. Although the site of bladder osteosarcoma was far from the bladder neck in all cases, transurethral surgery itself may be a risk factor for the development of bladder osteosarcoma. In this case, osteosarcoma appeared on the right wall, which is the same site as the initial urothelial carcinoma site, suggesting that stimulation by TURBT may have caused transformation of the bladder epithelium or stroma, resulting in the development of bladder osteosarcoma.

## Conclusion

We herein described a case of bladder osteosarcoma. Although osteosarcoma of the urinary bladder has an extremely poor prognosis, in the present case, in which it was detected at an early stage without subjective symptoms, treatment with TURBT alone resulted in long-term survival without recurrence.

## Supplementary Information

**Additional file 1. **Clinical characteristics of 38 cases of bladder osteosarcoma.

## Data Availability

Data sharing is not applicable to this article as no datasets were generated or analyzed during the current study.

## Abbreviations

TURBT: Transurethral resection of bladder tumor
CT: Computed tomography
MRI: Magnetic resonance imaging
VI-RADS: Vesical imaging reporting and data system
